# Reaching consensus: a review on sexual health training modules for professional capacity building

**DOI:** 10.15171/hpp.2018.01

**Published:** 2018-01-07

**Authors:** Zahra Karimian, Seied Ali Azin, Nasrin Javid, Marzieh Araban, Raziyeh Maasoumi, Shahrokh Aghayan, Effat Merghati Khoie

**Affiliations:** ^1^Student Research Committee, Shahroud University of Medical Sciences, Shahroud, Iran; ^2^Reproductive Biotechnology Research Center, Avicenna Research Institute (ACECR), Tehran, Iran; ^3^Faculty of Health, University of Technology Sydney, Sydney, Australia; ^4^Social Determinants of Health Research Center, Ahvaz Jundishapur University of Medical Sciences, Ahvaz, Iran; ^5^Department of Reproductive Health, Tehran university of Medical Science, Tehran, Iran; ^6^School of Medicine, Shahroud University of Medical Sciences, Shahroud, Iran; ^7^The Iranian National Center for Addiction Studies (INCAS), Institution for Risk Behavior Reduction, Tehran University of Medical Sciences, Tehran, Iran

**Keywords:** Sexual health, Professional capacity building, Systematic review, Iran

## Abstract

**Background:** Professional capacity building (PCB) is the focus point in health-related subjects.The present study was conducted to systematically review the existing sexual health training modules for health care providers.

**Methods:** The following keywords were used to search: training, education, professional capacity, practitioner, sexual health, skill education, module, course, package and curriculum.The term MESH is referred to Medical Subject Headings and the following databases were investigated: MEDLINE, EMBASE, PubMed, Cumulative Index to Nursing and Allied Health Literature (CINAHL), The Cochrane Library and Web of Science, Scopus, Google Scholar, SID,Magiran, and Iranmedex. All articles from 1980 to 2015 were extracted. Online modules were excluded. Considering that lesson plan was the basis of instruction, the modules were selected based on the characteristics of the lesson plans.

**Results:** A total number of 38 published training modules in the field of sexuality we redetermined. In total, more than half of the modules (58%) were designed for medical doctor sand allied health professionals and the remaining (42%) were for nurses and midwives. Almost all the modules (97%) were introduced and utilized in developed countries, and only 3% were disseminated in developing countries.

**Conclusion:** There are invaluable modules to build professional capacity in the field of sexual health. As a number of modules have been designed for nurses and midwifes, as the first-line health care providers, the use of these groups in sexual counseling and empowerment for sexual health is essential. No sexual health training program was designed in Iran. Therefore, designing such modules according to Iranian culture is strongly recommended.

## Background

Recent interest in consensus building to improve professional capacity for sexual health has its origins in the field of medical education. Medical education plays an important role in increasing the confidence and skills of healthcare providers to address the clients’ sexual health needs.^1^ There are also variations among medical educators in the degree of agreement necessary to finalize a decision on building sexual training capacity.^2^ Capacity building in health realm is the development of sustainable skills, organizational structures, resources and commitment to health improvement in all sectors to prolong and multiply health gains.^2^ In professional development or capacity building efforts, focus is on systemic approaches which may dramatically increase the quality of professional learning, as the final outcome.^3^

Professional capacity building (PCB) is the center of focus in health-related subjects and becomes seminal when sexuality is the subject matter.^2^ Sexual health, as an essential aspect of personal health, has influences on the overall health of an individual, throughout his/her life. Sexual health is a broad subject with many aspects including reproduction, contraception, sexually transmitted diseases prevention and healthy sexual relationships. These aspects are basically covered by both sexual health services and sexual health promotion interventions.^4^

The World Health Organization (WHO) has recommended the integration of sexual health into primary health care services, sexuality education and PCB.^5^ Sexual healthcare has been proposed by National Prevention Strategy and Healthy People 2020 to increase access to reproductive and sexual healthcare services.^6^ Literature has shown the high prevalence of sexual dysfunction in both developed and developing countries.^7,8^ Despite the high prevalence of sexual problems, sexual health have poorly been managed in the primary health care services which is likely due to the insufficient levels of skills, attitude and knowledge of health care providers in the field of sexuality.^9,10^

A previous study revealed that despite the willingness of patients to discuss sexual health with their care providers, the healthcare providers often have difficulties in addressing sexual health needs for reasons like shame and lack of sufficient training.^11,12^ Conway showed that general practitioners (GPs) would have the skills and the ability to manage patients with sexual health problems in primary care, only if they had received the appropriate training and support.^12^

Similarly, Nakopoulou et al clarified that sexual health assessment and intervention should be an integral part of nursing practice, which necessitates the introduction of modules into nursing curriculum in order to address the multidimensionality of sexuality.^13^ A study from Iran also reported that, in spite of providing a broad range of reproductive health programs in the health care system, comprehensive sexuality education has not yet been utilized.^14^ Many studies have highlighted the need for sexuality health education programs on the favor of clients and health care providers.^15,16^ Due to the sensitivity in the matters related to sexuality, cultural competence of health professionals is important when they provide their patients with sexuality-related health care. All societies need culturally sensitive sexual health modules from which Iran is not an exception.

Despite establishing a wide range of sexual and reproductive seminars and congresses in Iran,^17,18^ no modules is known for PCB in the field of sexual health care in the country. The relevant materials are not considered in the medical education curricula. There is only one credit course namely “Sexual Function and Dysfunction” for midwifery students in universities.^19^ No doubt, there is a great need to develop sexual health training modules based on the Iranian culture in the medical education curricula. Development of training modules specific to health professionals as well as building professional capacity in health-care systems are essential efforts to address primary sexual health needs. To do so, having a comprehensive understanding on the issue is required. However, there is a paucity of systematic appraisals of the existing modules with focus on sexual health. Therefore, the purpose of the present study was to critically review the existing sexual health training modules aiming at PCB.

## Materials and Methods

The present study was conducted to review the educational and training modules for PCB in the field of sexual health. The term ‘module’ in the present paper means a fractional part of a health professionals’ education experience. In an entire program, each class represents a module focused on a given subject.

### Search strategy

The following keywords were used to search: training, education, capacity professional, practitioner, sexual health, skill education, module, course, package and curriculum. The term MESH is referred to Medical Subject Headings and the following databases were investigated: MEDLINE, EMBASE, PubMed, Cumulative Index to Nursing and Allied Health Literature (CINAHL), The Cochrane Library, Web of Science, Scopus, Google Scholar, SID, Magiran, and Iranmedex. All articles from 1980 till 2015 were extracted. We also investigated some key organizations and associations including the Ministry of Health in different countries, and some university webpages, as well. We utilized various key combinations of words such as: “training” OR “education” AND “practitioner” OR “specialist” AND “sexual health”.

### Inclusion criteria

Packages, modules or programs concerning education in sexual health were identified and the target groups were also determined as health care professionals including physicians, midwives, nurses, and allied health professionals. The studies published in English were included. In order to prevent any insufficiency in the reported results of the investigated articles, the corresponding authors were contacted for further details.

PICO (patient, intervention, comparison and outcome) question was included in our review as P: health care provider I: sexology training and education C: routine medical education O: sexual health promotion.

### Exclusion criteria

The online modules were excluded considering that sexually-related modules need to be interactive, constructive and informative to reduce learners’ vulnerability and confusion.

### Assessment of modules

Considering the fact that the lesson plans are the basis for instruction, the selection criteria for the modules in our study were as follow^20^: Specification of duration, location, goal and content of the program, as well as the target group, teaching method, and assessment procedures. The search results were screened independently by 2 reviewers using a predefined inclusion criteria form. PRISMA (Preferred Reporting Items for Systematic Reviews and Meta-Analysis) checklist was applied for processing the steps of study.

## Results

**Figure 1:**
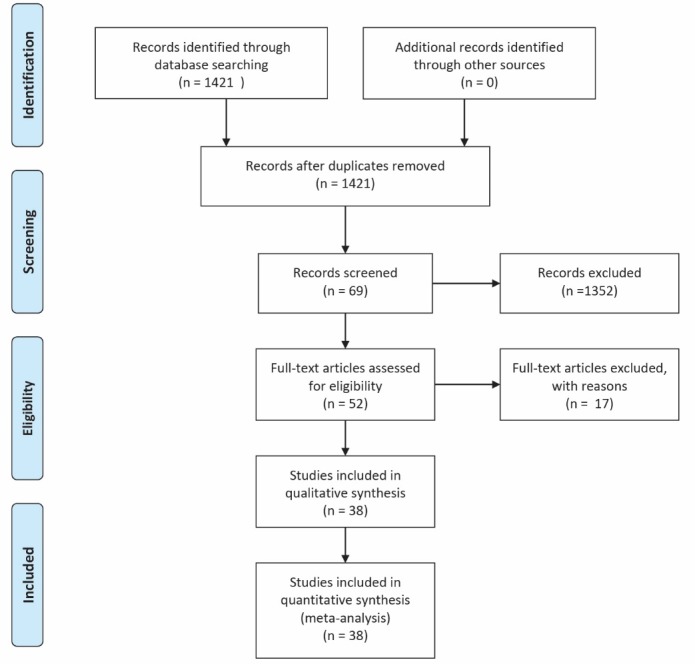
Flow diagram the dataset selection process.

Investigating the databases a total number of 1421 records were identified within which 52 modules were found. Thirty-eight out of 52 modules met the inclusion criteria. As mentioned above, 17 online modules were excluded due to different structures. Figure 1 clarifies the detailed processing of these datasets for final inclusion in this review.

The modules obtained in this study are summarized in Table 1. The modules were mostly (58%) for physicians, and about 73% had a lesson plan. A majority (97%) of the modules were introduced and utilized in developed countries and only 3% were disseminated in developing countries. About 18% were implemented in Australia. There were many theoretical and empirical subjects in these modules. A majority of the courses were implemented in a university with various times of duration. Assessment procedures in the modules included pre-test–post-test, solving case reports, as well as clinical and practical functions.

The modules’ characteristics varied by countries. However, there were some similarities: all the modules included anatomy and physiology of male and female reproductive organs, sexually transmitted diseases, methods of contraception, and counseling in sexual health. The main focuses of many programs (28.9%) were contraception methods and sexually transmitted diseases. Content of the modules are summarized in Table 2.

## Discussion

Through the present review we found invaluable and efficient training modules for PCB in the field of sexual health. A number of modules were designed for nurses and midwifes (Table 1). According to the findings, it seems that nurses and midwifes are considered to be the first-line healthcare providers who play an important role in providing sexual services to patients or clients. The role of practice nurses (PNs) is essential in sexual health care as their role in some areas like chronic disease management and immunization.^15^ Moreover, nurses are considered as the first-line health care providers even in the treatment of sexually transmitted infections which are relatively new concepts in developed societies such as the United Kingdom.^15^

Some of the modules were also found targeting midwives (Table 1). This may be considered as a strong point for these modules because the role of midwifery profession in women’s sexual health promotion has been recognized worldwide. Nematollahzadeh et al, in a systematic review pointed out to the role of a midwife in women’s sexual health promotion as undeniable, and concluded that midwives can have a key role in promoting sexual health.^59^

It has been documented that those sexual modules with focus on preventing unintended pregnancies or STD/HIV infection are of important consideration.^60^ It may be considered as another strength for this modules because appropriate sexual education programs should cover STDs prevention and unwanted pregnancies.^61^

The most of modules’ contents reviewed in our study had focus on the biology of sex, only. This may be a weakness for these training programs considering that effective sexual training programs should have a good coverage on the psychological, social and biology aspects of sexuality.^61^ Offering biological aspects of sexuality as the only content of a module (for example CASH module and SH&FPA module) might reduce the value and depth of the module in a courses.

Most of these modules are being hold in developed countries. Despite the great need for sexual-health services in developing countries, especially for women regarding topics of family planning and screening for STDs, a small number of sexuality training modules is established in these countries to educate and train the health professionals. With the hope to improve health outcomes, the department of reproductive health in the WHO has had some community based initiatives especially in such countries.^3^

As a limitation for our study, the effectiveness of these programs was not evaluated. The effectiveness of these modules may be evaluated through assessing the changes in knowledge, attitude and practice of health care providers as well as the changes in the rate of sexual problems.

Formative evaluation should be taken place before or during a project’s implementation with the aim to improve the project’s design and performance. Lack of formative evaluation may be a notable limitation for implementing these modules.

To build professional capacity, sexuality training modules need to be evidence-based and systematically evaluated, culturally sensitive, and skillfully implemented. It is, however, challenging to deliver culturally sensitive and appropriate sexuality-related health care in a context without training modules integrated in medical education programs. Merghati Khoie et al manifested that religion is an important aspect of cultural foundations of sexuality among Muslim Iranian women.^62^ As a sexologist, she argued that professionals working in the fields of health and sexuality need to be sensitive and apply culturally appropriate therapies for Iranian population.^63^

In Iran, midwives have a key role in promoting reproductive health of women in various settings.^14^ in clinical settings, while educating midwives on women health issues, sexual problems are generally discussed, as well. However, midwives seem to not have enough knowledge and skills in sexual consultation.^14^ This lack of knowledge and skill among healthcare providers in Iran urges the need for development and implementation of specific sexual health modules for capacity building among Iranian care providers. It is also recommended to integrate sexual health consultation courses into medical students’ curricula, as they are at the first line of encountering patients with sexual problems.

Based on this review, we suggest that a sexual module should undergo validity process to ensure users from the best selection of both professional goals and the ways to achieve the goals. There was a scarcity of modules designed to improve sexual health of specific groups of patients. This might help professionals to tailor their education targeted at those groups to enhance effectiveness of sexual programs.

## Conclusion

There are invaluable and efficient training modules for PCB in the field of sexual health. Considering the small number of developing societies employing such modules to educate and train their health professionals, the need to design sexuality modules in these countries is outstanding. A number of modules in different countries have been designed for nurses and midwifes as the first-line healthcare providers for communities. Therefore, more employment of these groups of health professionals in sexual health counseling and empowerment is suggested. Moreover, family health care providers should be focused as one of the main target groups of health professional while designing sexual health modules. To promote PCB of health care providers, a culturally adaptive module with a clear and valid content, especially in developing countries, seems to be necessary.

## Implications for Practice

Sexual health practitioners may greatly benefit from the results of this study while designing needs assessment and/or designing, implementation and evaluation of sexual health promotion programs, especially in the contexts of developing countries like Iran. Our results might also be applied by health authorities for developing a sexual module compatible with the Iranian culture.

## Ethical approval

This review was approved by Ethic Committee of Shahroud University of Medical Sciences in 2015 (The ethical code: IR.SHMU.REC.2015.44).

## Competing interests

Authors declare no conflict of interest.

## Authors’ contributions

All authors were involved in drafting the article or revising it critically for important intellectual content, and all authors approved the final version to be submitted for publication. EMK had full access to all of the data in the study and takes responsibility for the integrity of data and the accuracy of data analysis. ZA, EMK and SAA participated in study conception and design. Acquisition of data was done by ZA, MA, SA and finally analysis and interpretation of data was conducted by ZA, NJ, RM and EMK.

## Funding

Funding for this study was provided by Shahroud University of Medical Sciences.

## Acknowledgments

We thank all those who help us to design and conduct this study.

**Table 1:** Characteristics of modules

**No.**	**Type**	**Name (Reference)**	**Date**	**Year**	**University**	**Country**	**Target group**	Level*
‏1	‏Module	‏CASH (Contraception and Sexual Health) ^21^	‏September each year	‏2015	‏South Wales Prifysgol	‏Britain	‏Nurses and midwives	‏6
‏2	‏Course	‏Shine SA (sexual health information networking and education south Australia)^22^	‏Over 5 consecutive days or the 6 modules	‏2015	‏South Australia	‏Australia	‏Nurses and Midwives	‏
‏3	‏Certificate course	‏SH&FPA: Certificate in Sexual and Reproductive Health for Medical Practitioners ^23^	‏5-day theory	‏2015	‏Western Australia	‏Australia	‏Medical practitioner	‏7
‏4	‏Module	‏Sexual Health^24^	‏For 5 weeks at 2.5 days per week	‏Academic year 2014-15	‏London School of Hygiene & Tropical Medicine	‏England	‏Students to issues involved in working in the area of sexual health, whether as researchers, practitioners or evaluators	‏7
‏5	‏Course	‏Sexual and Reproductive Health^25^	‏-	‏-	‏University of the West Scotland	‏Scotland	‏Nurses and midwives	‏‏-
‏6	‏Programme	‏Sexual Health, BSc Hons (Top up)^26^	‏-	‏-	‏University of Greenwich	‏England	‏Nurse or midwife who wishes to work in sexual health	‏‏-
‏7	‏Module	‏Module of sexual health^27^	‏-	‏2013-2014	‏University of London	‏England	‏Students	‏7
‏8	‏Certificate course	‏New Zealand family planning certificate in contraception and sexual health^28^	‏4-day course	‏‏-	‏New Zealand	‏New Zealand	‏Midwives primary healthcare professionals	‏‏-
‏9	‏Package	‏The FSRH Diploma training package,^29^ SRH: Faculty of Sexual and Reproductive Healthcare	‏‏-	‏‏-	‏Royall collage of nursing	‏UK	‏Is for doctors (DFSRH) and nurses (NDFSRH)	‏‏-
‏10	‏Course	‏Family planning Queensland: FPQ Introduction to Sexual & Reproductive Health Theory ^30^	‏‏-	‏‏-	‏Australia, Queensland	‏Australia	‏Nurses and Allied Health Professionals	‏‏-
‏11	‏Course	‏Clinical Aspects of Sexual and Reproductive Health Course^31^	‏3 days	‏2016	‏Australia Queensland	‏Australia	‏Nurses and health workers	‏‏-
‏12	‏Course	‏HIV, STIs and Sexual Health - restructured in 2015^2^	‏‏-	‏2015	‏The university of Sydney	‏Australia	‏Professional in medicine	‏‏-
‏13	‏Courses	‏Sexual and Reproductive Health Nuts and Bolts of Sexual Health^33^	‏3-day course	‏2015	‏Western Australia	‏Australia	‏Nurses and other health professionals	‏‏-
‏14	‏Program	‏Family Planning NSW Reproductive and Sexual Health - Clinical Accreditation Program (RSH-CAP)^34^	‏8 sessions (4 days) of clinical placement with no more than 6 sessions (3 days) at one clinic	‏‏-	‏ New South Wales	‏Australia	‏Nurses/midwives	‏‏-
‏15	‏Course	‏Graduate Certificate in Sexual Health^35^	‏‏-	‏‏-	‏University of Melbourne	‏Australia	‏At health practitioners, particularly general practitioners or nurses	‏‏-
‏16	‏Module	‏Sexual & reproductive health^36^	‏‏-	‏2014-2015	‏Edinburge Napier university	‏Scotland	‏‏-	‏‏-
‏17	‏Module	‏Sexual Health Module^37^	‏9 days theory and 25 days clinical	‏2014-2016	‏Middlesex University London	‏England	‏Registered nurses, health visitors and midwives	‏6,7
‏18	‏Module	‏Sexual and reproductive health care (e-SRH) modules^38^	‏‏-	‏‏-	‏Royall collage of nursing	‏UK	‏For nurses	‏‏-
‏19	‏Module	‏Contraception and Sexual Health Awareness ^39^	‏6 full days	‏2015-2016	‏University of Nottingham	‏UK, China, Malaysia	‏Health and social care professionals.	‏7
‏20	‏Module	‏Sexual health and well-being across the lifespan ^40^	‏5 days	‏2016	‏Kingston University	‏England	‏Healthcare professional	‏7
‏21	‏Module	‏Theory of Contraception and Sexual Health ^41^	‏Semester 1 40 days	‏‏-	‏University of Wolverhampton	‏UK	‏Midwife	‏5 or 6
‏22	‏Module	‏Sexual Health Module^42^	‏5 days	‏2015	‏University of Surrey	‏England	‏Students	‏7
‏23	‏Certification	‏Sexual Health and Treatment^43^	‏‏-	‏‏-	‏Metabolic medical institute	‏Malaysia	‏Provider	‏‏-
‏24	‏Course	‏PG Cert Sexual Health^44^	‏‏-	‏‏-	‏University of Bradford	‏England	‏‏-	‏7
‏25	‏Module	‏STIs and Screening Interventions in Sexual Health^45^	‏‏-	‏‏-	‏De Montfort university	‏England	‏Nurses and allied health professionals	‏‏-
‏26	‏Module	‏Contraception and Reproductive Sexual Health^46^	‏‏-	‏2015-2016	‏University of cumbia	‏USA	‏Practitioners	‏6
‏27	‏Module	‏Developing Skills in Contraception and Reproductive Sexual Health Care^47^	‏‏-	‏2015-2016	‏University of Campus Suffolk	‏USA	‏Nurses, midwives and health visitors	‏6
‏28	‏Course	‏Contraception and Sexual Health Foundations^48^	‏6 days	‏2015	‏University of York	‏England	‏‏-	‏6 and 5
‏29	‏Module	‏Contraception and Sexual Health - Praxis CPPD ^49^	‏5 taught days	‏2014	‏City University London	‏England	‏‏-	‏‏-
‏30	‏Module	‏Understanding Contraception & Reproductive Sexual Health ^50^	‏5 days	‏‏-	‏Anglia Ruskin University	‏UK	‏Professional	‏6
‏31	‏Course	‏MSc Sexual and Reproductive Health^51^	‏Full-time: 1 year	‏‏-	‏Queen Margaret University	‏Mussel Burgh, East Lothian	‏Health professionals	‏‏-
‏32	‏Module	‏HIV & Sexual Health^52^	‏‏-	‏‏-	‏Bart and the London School of medicine	‏England	‏Students, doctors	‏‏-
‏33	‏Course	‏M.Sc. Sexual Health^53^	‏‏-	‏2016	‏Glasgow Caledonian University	‏UK	‏‏-	‏‏-
‏34	‏Course	‏Professional course Sexual health^54^	‏‏-	‏2015-2016	‏University of the west of England	‏England	‏Nursing and midwifery	‏‏-
‏35	‏Course	‏Short Course Issues in Contraceptive and Reproductive Sexual Health^55^	‏10 days	‏2012	‏Anglia Ruskin University	‏UK	‏Professional	‏‏-
‏36	‏Short course	‏Sexual & Reproductive Health master module- Short Course^56^	‏‏-	‏‏-	‏Queen Margaret University, Edinburgh	‏Scotland	‏Professional	‏‏-
‏37	‏Module	‏IGWG Gender, Sexuality and HIV Training Module^57^	‏2 days	‏	‏United States Agency for International Development (USAID)	‏USA	‏	‏-
38	Course	Sexual & Reproductive Health Training for Doctors 2015^58^	6 days	2015	SHFP ACT Sexual health and family planning ACT University Avenue, Canberra	Australia	Doctors	-

* Level 7: postgraduate level; Level 6: undergraduate level.

**Table 2:** Content of modules

**Name of module**	**About the modules**	**Content**
CASH module (Contraception and Sexual Health)	CASH is the name for the contraception, sexual health and reproductive health and gynecology services in Greenwich	Anatomy and physiology of the male and female reproductive tracts Fertility awareness and natural family planning Human sexuality Counseling Sexual health strategy Service aims, provision and integration
Shine SA (sexual health information networking and education South Australia)	Shine SA is a leading not-for-profit provider of primary care services and education for sexual and relationship wellbeing in South Australia	Core concepts of sexual health Reproductive health Sexually transmitted infections and blood-borne viruses Women’s sexual health Men’s sexual health Sexual health counseling
SH&FPA: Certificate in Sexual and Reproductive Health for Medical Practitioners	This certificate course covers sexual and reproductive health issues for both women and men at a post graduate level in Australia.	Female and male reproductive physiology Contraception: information on different contraceptive options, both hormonal and non-hormonal, and management of problems associated with their use. Management of unplanned pregnancy, including medical, psychosocial and legal aspects Diagnosis and management of sexually transmitted infections Cervical and breast screening Menopause management Management of couples with subfertility Office gynecology: menstrual problems, vulvar disorders, fertility etc Men’s sexual health: office urology, male sexual difficulties
London School of Hygiene & Tropical Medicine: Sexual Health Module	The London School of Hygiene & Tropical Medicine is a world-leading center for research and postgraduate education in public and global health.	The nature of sexual behavior: theoretical and empirical issues: definitions of normal, diversity and conformity, and implications for sexual health. The regulation of sexual conduct; Historical and social science approaches to the study of sexual behavior (i.e. variations through time and across societies). Trends in sexual mores and their implications for public health. Influences on sexual attitudes and lifestyles – psychological, biological, cultural, religious, political, technological, etc; Political aspects of sexual health; treatment of sexual issues in society, implications of stigma, sensitivities and taboos for practice of sexual health medicine and provision of services. Communication about sexual matters. Gender issues in sexual health; Researching sexual behavior: pitfalls and possibilities; appropriate and inappropriate methodologies; examples of qualitative and quantitative research; Public health priorities in sexual health. Designing, executing and assessing sexual health interventions.
Sexual Health, BSc Hons (Top up) program:	This program in university of Greenwich is for those who have a diploma in higher education and are a registered nurse or midwife who wishes to work in sexual health.	To develop a knowledge base which will enable the student to select and apply appropriate knowledge to her/his practice in sexual health To promote understanding of concepts related to sex, sexualities and sexual health, including the negative effects related to prejudice and stigma To enhance professional accountability and ethical decision making within the inter-professional context
Module of sexual health: University of London	This module is in University of London about sexual health	Conceptual and theoretical aspect of sexual health Risk and vulnerability Intervention to improve sexual health Measuring and assessing of sexual health status
The FSRH Diploma training package: FSRH: Faculty of Sexual and Reproductive Healthcare	FSRH is a faculty of the Royal College of Obstetricians and Gynecologists established on March 26, 1993 as the Faculty of Family Planning and Reproductive Health Care	Global aspects of sexuality and health Sexual identity and sexual development Sexuality issues throughout the lifespan Cultural perspectives Impact of disability and illness on sexuality Talking sex and sexuality Legal issues Sexual violence Sexually transmissible and reproductive tract infections Male reproductive health Female reproductive health Contraception Pregnancy choices Clinical Aspects of Sexual and Reproductive Health Course Contraception, unplanned pregnancy and STIs. Blood borne viruses Issues in sexual health service delivery
Family Planning NSW Reproductive and Sexual Health – Clinical Accreditation Program (RSH-CAP)	Family Planning NSW is the state’s leading provider of reproductive and sexual health services	Recognize and evaluate factors that influence an individual’s reproductive and sexual health choices competently deliver reproductive and sexual health care reflect on own practice and update according to evidence based practice standards
Sexual Health and Family Planning ACT (SHFPACT): Sexual & Reproductive Health Training for Doctors 2015:	SHFPACT (Sexual Health and Family Planning ACT) is a not-for-profit, non-government, membership-based organization, and is a member of Family Planning Alliance Australia - a network of independent, state-based reproductive and sexual health organizations - and the International Planned Parenthood Federation (IPPF).	The course is structured in three modules: Module 1: Theory component (face-to-face training workshops) include: Contraceptive technology Women health and STIs Men sexual health across the lifespan Gynecological issues Sexual health counseling Legal issues in sexual health Module 2: Assessment (Written & Role Play Exam on all topics covered in course) Module 3: Clinical Attachment
Sexual & Reproductive Health master module - Short Course: Queen Margaret University, Edinburgh	This course is designed to provide students with an overview of historical and current issues and debates in the area of sexual and reproductive health, with special attention to power relations, gender and a global economic perspective	Constructions of sexuality -conferences and contestation: historical development of conceptualizations of sexual and reproductive health Rights-based approaches Theories about the relationships between development, population growth and reproductive health and how these inform SRH programs - Politics and economics of sexual and reproductive health interventions (e.g., concerning assisted reproductive technologies) Current sexual health (e.g., STIs) and reproductive health issues (infertility, abortions, maternal mortality, FGM) Gender-based violence (including medical violence, e.g. forced sterilizations, forced abortions, harmful treatments of fertility) Sexual and reproductive health across the life cycle (special attention for youth and elderly) Sexual and reproductive health and men Sexual and reproductive health promotion
Short Course Issues in Contraceptive and Reproductive Sexual Health Anglia Ruskin University, United Kingdom:	This module is designed to introduce and develop an understanding of contraceptive and sexual health care	Contraceptive methods Sexually transmitted diseases Human sexuality Sexual development Legal and Ethical Issues e.g. Fraser guidelines Psychosocial influences e.g. drugs, alcohol, media Cultural issues Political issues Sexuality, Disability and Special Needs Local and national Provision Access to Services Facilitating informed choice Lifespan approach to contraception and sexual health e.g. adolescents, perimenopause, menopause
Professional course Sexual health: University of the west of England		Knowledge and understanding: Review of anatomy and physiology of the male and female genital tract Methods of contraception Pathophysiology of the male and female genital tract Disability and reproductive sexual health Intellectual skills: Men's and women's health issues Health education and promotion issues in relation to reproduction and sexual health Sexuality Gender, cultural and sociological issues Legal and ethical issues in reproductive and sexual health Government Sexual Health Strategy. Subject/professional and practical skills: Contraception across the reproductive age span Managing the clinic environment Transferable skills: Professional issues in reproductive and sexual health Personal development, including application of appropriate communication skills
Glasgow Caledonian University — M.Sc. Sexual Health:		PgC Sexual Health (two core modules): Sexuality and Sexual Health; Reproductive Health. PgD Sexual Health: two core modules and the Advanced Research Methods double module. MSc Sexual Health: two core modules; a double leadership module; Research Dissertation; and two modules from any recognized masters program.
Understanding Contraception & Reproductive Sexual Health, Anglia Ruskin University	The mission of the course of university is they are exceptional and imaginative in the advancement of knowledge and education of students	Contraceptive methods Sexually transmitted diseases Human sexuality/sexual development Legal & ethical issues (e.g. Fraser guidelines) Psychosocial influences (e.g. drugs, alcohol, the media) Cultural issues Political issues Sexuality, disability & special needs (local & national provision) Access to services Facilitating informed choice Lifespan approach to contraception & sexual health (e.g. adolescents, perimenopause, menopause
University of Cumbria: Contraception and Reproductive Sexual Health	This module is designed to prepare practitioners to be competent in the safe and effective administration of contraception and associated sexual health promotion	Competently take a sexual and contraceptive history to inform a holistic client assessment. Demonstrate a sound knowledge and understanding of and apply legal, professional and ethical frameworks for contraceptive and reproductive sexual health advice Critically analyse your development and application of detailed knowledge of methods of contraception and issues surrounding their administration. Critically evaluate and apply evidence-based health promotion and health prevention approaches to reduce risk and maintain sexual and reproductive health. Demonstrate a sound knowledge of local and national services and referral pathways to enable joined up care and facilitate choice.
Sexual Health Module: University of Surrey (in England)	This module aims to develop and enhance a student’s knowledge and skills in relation to sexual health	Demonstrate a clear understanding of the social context in which sexuality and sexual health is experienced as well as critically examine the policy context including historic influences. Examine the images and media construction, culture and demographic influences on sexual health in contemporary Britain and immunized their relationship to public perception. Demonstrate evidence based knowledge of contraception, sexually transmitted infections, blood borne viruses, gynecology, sexuality & psycho-sexual issues. Discuss medico-legal & ethical issues associated with sexual health care and critically analyze their impact on practice with specific reference to consent and confidentiality. Demonstrate a broad understanding of the needs of specific groups such as teenagers and other high risk or vulnerable groups.
Sexual health and well-being across the lifespan course: Kingston University	This course is in Kingston University and the aim of module is promotion of sexual health	Critically examine and discuss sexuality, sexual orientation, sexual and psychosexual issues in relation to health. Critically discuss the concept of ‘safer sex’ and sexually transmitted infection, with reference to communication skills and strategies when dealing with client groups. Critically examine and discuss attitudes relating to gender, culture, ageism, heterosexuality and homosexuality. Critically analyze the ethico-legal aspects around sexual health. Critically discuss the implications of current policy in relation to sexual health from an international, national and local perspective.
Contraception and Sexual Health Awareness: University of Nottingham	This university have education for Health and Social Care Professionals	Factors influencing the sexual health of clients, patients and their significant others Assessment, Planning, Implementation and Evaluation of strategies for meeting sexual health needs of clients Exploration of emotional, cultural and social influences on sexuality, sexual expression, sexual health and ill health Exploration of choices for effective contraception for males and females of various ages and in various situations Examination of the effects of common sexual health problems, including sexually acquired infections, on patients, clients and their significant others
Sexual Health Module: Middlesex University London	This course will enable you to develop advanced knowledge and skills in sexual health	Anatomy and physiology of the male and female reproductive system Testing, diagnosis , treatment and management of STD Sexual health polices Psychosocial perspectives Adolescence and sexual identity Reproductive sexual health issues Female genital mutilation Current and developing trend in the provision of sexual health care and services
Sexual & reproductive health module: Edinburg University	Edinburgh Napier University has been working on developing and enhancing the information we publish on the Module Catalogue. This particularly affects the Learning, Teaching (Student Activity) and Assessment sections	Pre course work is revision of the physiology of the normal menstrual cycle and the normal male and female genital anatomy. Course work will involve input from specialist lecturers & practitioners and will include: Methods of contraception Communication in sexual health Impact of diversity on sexual health across the lifespan Infertility and unplanned pregnancy Sexually Transmitted Infections and non STI’s encountered in non-specialist settings Collaborative working
